# Bibliometrics and Visual Analysis of the Research Status and Trends of Postpartum Depression From 2000 to 2020

**DOI:** 10.3389/fpsyg.2021.665181

**Published:** 2021-05-24

**Authors:** Xue Bai, Zixuan Song, Yangzi Zhou, Xiaoxue Wang, Yuting Wang, Dandan Zhang

**Affiliations:** ^1^Department of Health Management, Shengjing Hospital of China Medical University, Shenyang, China; ^2^Department of Obstetrics and Gynecology, Shengjing Hospital of China Medical University, Shenyang, China

**Keywords:** bibliometrics, postpartum depression, PPD, knowledge map, visualization analysis

## Abstract

The purpose of this study was to evaluate the international scientific output on postpartum depression (PPD) research during 2000–2020 through a bibliometric analysis and to explore research hotspots, frontiers, and trends in the field of postpartum depression. We searched the Web of Science Core Collection for publications on postpartum depression published between 2000 and 2020. CiteSpace, gCluto, and other software applications were used to analyze the data by year, journal, and country. A total of 2,963 publications were retrieved and 96 countries or regions published related papers. The United States had the largest number of published papers and the highest betweenness centrality, which is the dominant position in the field of postpartum depression. A total of 717 journals published papers, with the Archives of Womens Mental Health ranked first in terms of volume and betweenness centrality. In this study, 31 high-frequency main MeSH terms/subheadings were selected. The high-frequency MeSH terms were clustered into six categories: an overview of depression-related research, diagnostic and screening scales for postpartum depression, epidemiological investigation into postpartum depression, treatment and drug selection for postpartum depression, psychological research on postpartum depression, and etiology, physiopathology, complications, genetics of postpartum depression. Finally, we used strategic diagram to analyze research trends in postpartum depression. This study has identified a continuous significant increase in the publication of PPD articles. Currently, the etiology, physiological pathology, intervention and treatment of complications on PPD are immature, which provides reference for the trend of obstetric psychology.

## Background

Roland (1950) first proposed the concept of postpartum depression (PPD) in 1950. According to the American Classification of Mental Disorders and the Diagnostic and Statistical Manual of Mental Disorders (DSM-5), the onset time of PPD is within 4 weeks after delivery, but the definition of the onset time of PPD refers to a period of between 1 day and 12 months after delivery. Due to the differences in diagnostic criteria, study design, study time, sampling method, sample source, and socio-demographic data, the prevalence of PPD in the survey varied greatly. The prevalence of PPD was 7–40% in Western developed countries (Liu and Tronick, 2013) and 3.5–63.3% in Asian countries (Simhi et al., 2019). The internationally recognized prevalence of PPD was 10–15% (Shorey et al., 2018). Studies have also found that PPD is increasing over time.

The harm caused by postpartum depression is mainly manifested in two aspects (Bodnar-Deren et al., 2016). First, parturients with PPD may be vulnerable to self-harm and suicide. After the first attack of PPD, more than half will experience another attack within the next 5 years and a third may even have another attack within a year. With the increase in the number of recurrences, the risk of further recurrence increases and the difficulty of treatment also increases. Second, PPD patients may have thoughts of harming their infants and present infant punishing behaviors and mother-infant connection disorders. This can lead to intellectual, emotional, and personality development disorders in their children, which increases the risk of violence among young people and places a heavy burden on families and societies. Therefore, postpartum depression, as a research hotspot, has attracted extensive attention.

PPD, in its complex and varied clinical manifestations and individual differences, includes a core syndrome (emotional distress, loss of interest and feelings of happiness, increased fatigue and decreased activity, and energy reduction), elements of a mental disorder (anxiety and reduced concentration, poor self-assessment and a lack of confidence, the concept of sin, low self-worth, pessimism, suicide ideation or ideas/behavior that could harm the infant, violence, and psychotic symptoms), and physical symptoms (sleep disorders, loss of appetite and weight loss, decreased libido, and non-specific physical symptoms) (Putnam et al., 2017). The diagnosis of PPD is mainly based on a combination of symptomatic characteristics and the course of the disease. There are no objective physical, laboratory, or exact imaging examinations, and no specific examination items. Results from evidence-based medicine has shown that comprehensive treatment, whole-course treatment, and graded treatment are recommended for the treatment of PPD in order to ensure the safety of the mother and infant (Stewart and Vigod, 2019). However, the selection of antidepressants is still controversial, and the hierarchical management mode of PPD is still in an exploration stage.

The knowledge map is a type of research method that visualizes the core structure, development process, frontier field and the whole knowledge framework of a discipline. The research methods are diverse, including bibliometric analysis, citation and co-citation analysis, co-word and co-occurrence analysis, multivariate statistical analysis, social network analysis, etc. (Gu et al., 2020). Bibliometrics has a wide range of applications and plays an important role in the theoretical and practical research of information science. Bibliometrics can be used to clarify the characteristics of literature, analyze and estimate the development trend of a certain field, sorting and statistical data. For researchers, with the increase of scientific literature, the time for scientific researchers to consult literature is greatly extended, and it is difficult to complete and identify relevant literature in the field to be studied by relying on original methods, which seriously affects the efficiency of scientific research. With the method of bibliometrics, we can quickly understand the background of literature and grasp the development trend and direction of the subject.

In recent years, depression-related bibliometrics research has been gradually carried out. Wang X. et al. (2020) used bibliometrics to analyze the data of annual published papers, major research institutions, core authors and research topics, and then identified the core authors and core teams, analyzed the current research direction of RA-related depression, and predicted new research topics. Hammarström et al. (2009) employed bibiometrics to explain the scientific quality of explanatory biological models associated with gender-related depression. At present, the number of studies on PPD is increasing, but the research directions are different and lack of proper control, so the research results are uncertain. For example, diagnostic criteria for PPD, prenatal intervention for PPD, and systematic management of PPD have not been established. Now, there is a lack of analysis on these related research directions, research depth and research hotspots. This study adopted bibliometrics method, combined with clustering research hot spots and strategy diagram-based research frontier analysis, to analyze the research hot spots, frontier and key literatures of postpartum depression, and predict the research trend in this field, in order to provide a comprehensive and intuitive reference for subsequent researchers.

## Data and Methods

### Data Collection and Search Strategy

The original data for this article came from the Web of Science core collection database including SCI-EXPAND‵SSCI. The search strategy of our study was TI = (“postpartum depressi^*^” OR “postnatal depressi^*^” OR “post-partum depressi^*^” OR “post-natal depressi^*^” OR “post partum depressi^*^” OR “post natal depressi^*^”) OR AK = (“postpartum depressi^*^” OR “postnatal depressi^*^” OR “post-partum depressi^*^” OR “post-natal depressi^*^” OR “post partum depressi^*^” OR “post natal depressi^*^”), and restricted the publication date to between January 01, 2000 and December 31, 2020. The manuscript type was “Article” and we checked the relevance of the results. The relevant content was set to “full record,” the file format was set to “plain text,” the records were exported in batches and named “download1–6.txt” and saved in the same folder. For the subsequent analysis, two researchers manually searched the PubMed database, downloaded and proofread the literature of the above search results, and saved the results as XML files.

### Visual Knowledge Map Analysis and High-Frequency Main MeSH Terms/Subheadings Biclustering Analysis

The Citespace (Wang Z. et al., 2020) software reflected the internal connections of the research topic through the differences in the size and color of the “ring.” An “annual ring” represents a node. The thickness of the “rings” is proportionate to the frequency of occurrence of the node's content. The connections between nodes represents the co-occurrence relationship between nodes and the thicker the connection, the closer the relationship between the nodes. The frequency and betweenness centrality of the nodes take into account the status and communication power of the nodes in the knowledge map. In this study, Citespace 5.0.R1.SE was used to import text documents into the software to draw the required visual knowledge map. The parameters were set as follows: the time segment was from 2000 to 2020, the time span was 1 year, the node type was “cited-journal” and “country,” the default selection criteria was “Top N = 50.” Pruning selected “Pathfinder” and “pruning sliced networks” and visualization selected “cluster view-static” and “show-merged network” to generate a visual knowledge map.

The download 1-6 from Web of Science core collection database was imported into the BICOMB (bibliographic item co-occurrence matrix builder) software, independently developed by Professor Cui Lei (Li et al., 2015) of the China Medical University, to extract the data. We used Bradford's Law and and Dr. Egghe (Brookes, 1969; Leimkuhler, 1976; Egghe, 1990; Venable et al., 2016) to calculate the Bradford dispersion coefficient and the number of core journal. The Institute for Scientific Information (Philadelphia, PA, USA) developed the impact factor (IF), which was published annually in the journal citation reports (JCR) section of the science citation index (SCI). We itemized the IF value of JCR in 2020 (Garfield, 1979). The XML format file downloaded from the PubMed database was imported into the BICOMB (Li et al., 2015). The key field was set as the main MeSH terms/subheadings. The main MeSH terms/subheadings was mined and analyzed, while the frequencies of all the main MeSH terms/subheadings were extracted and counted. In this study, MeSH terms, with a frequency of occurrence ≥31 times, were defined as high-frequency MeSH terms by H index. Subsequently, 31 high-frequency MeSH terms were extracted from the included publications to represent the research hotspots of PPD.

This study used gCluto 1.0 (graphical clustering toolkit) software (Rasmussen and Karypis, 2004) of the University of Minnesota to import high-frequency main MeSH terms/subheadings into the co-word matrix. The clustering method selected Direct. Other parameter Settings showed similarity functiong as cosine and criterion function as I2. In order to select the optimal number of clusters, we took the minimum average similarity between classes (ESim) and the maximum average similarity within classes (ISim) as the optimization indexes. We performed a biclustering visualization analysis, drew a mountain visualization and visualization matrix, and calculated the number of clusters. The mountain visualization is a three-dimensional clustering map generated by a multidimensional scaling method. The color of the peak of the mountain visualization is inversely proportional to the standard deviation within the clusters. Red represents a small standard deviation and blue represents a large standard deviation. The height of the mountain is proportional to the similarity within the cluster. The steeper the mountain, the greater the similarity within the cluster. The volume of the mountain is directly proportional to the number of MeSH terms in the cluster. The distance between the peaks reflects the correlation between the clusters. The clusters with strong correlations has the possibility of gathering and superimposing. The value of the original data of the matrix in the visualization matrix was replaced by color. The depth of the color represented the frequency of MeSH terms. The darker the color, the higher the frequency. The clusters in the rows of the matrix represented the clusters of high-frequency MeSH terms/subheadings, and the clusters in the columns represented the PMID clusters corresponding to the source documents.

### Strategic Diagram Analysis

The strategic diagram is used to describe the internal relations and the interaction between a certain research field, which was proposed in 1988. Using the co-word matrix of high-frequency MeSH terms, the strategic diagram is established to further analyze the research and development stage of the clustering results of postpartum depression. The two-dimensional coordinates drawn with centrality and density as parameters are strategic diagram. Among them, the X axis represents the centrality, indicating the intensity of the interaction between domains, and the calculation of the centrality is calculated by the strength of the links between all the MeSH terms in a cluster and other MeSH terms in other clusters, that is, the sum of links between clusters. The Y axis represents the density, indicates the internal connection strength of a certain field, calculates the number of simultaneous occurrence of each MeSH terms in the same literature in this cluster, and obtains the density of this cluster by calculating the average value of these internal links. The horizontal axis and vertical axis divide the two-dimensional space into four quadrants. The first quadrant (high density and centrality) is internally connected and is at the center of the research network. The second quadrant (high density, low centrality) is closely linked within the subject area, the topic is clear, and some research institutions are conducting formal research on it, but it is at the edge of the whole research network. The third quadrant (low density and centrality), which shows that its internal structure is relatively loose, the research is not yet mature, and it is at the edge of the whole research field. The research is in an immature state. The fourth quadrant (high centrality, low density) the research subject domain structure of this quadrant is relatively loose, the research is not yet mature, it is closely integrated with other studies in the network, and there is space for further development in this field. It is of great potential importance in the whole research field. According to the coordinate quadrant of the calculated value, we not only speculated on the subject theme, but also analyzed the research status and development trend of the research topics related to postpartum depression.

## Results

### Distribution

**Figure 1:**
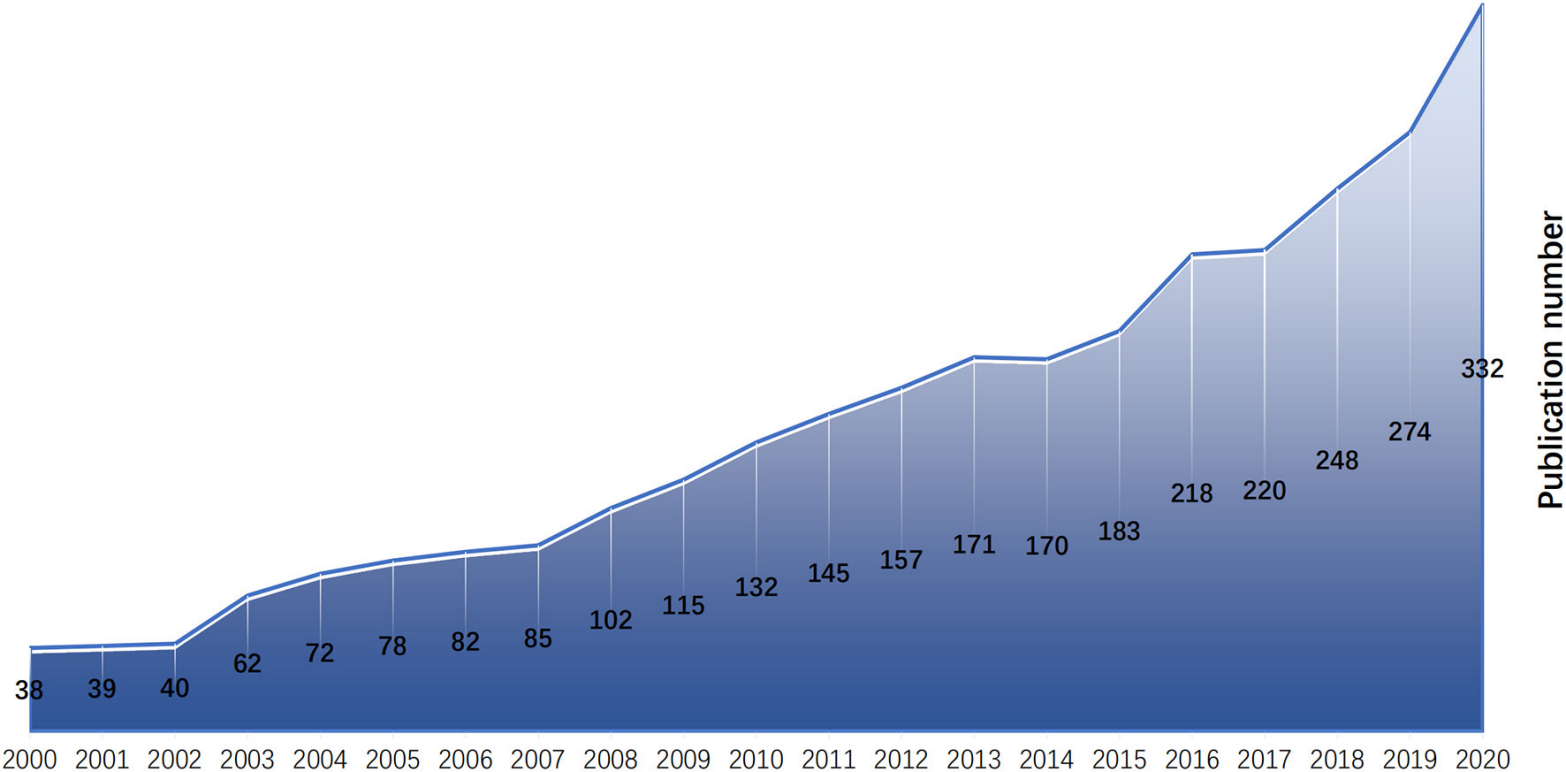
Growth of scientific production on PPD from 2000 to 2020.

According to the search strategy and manual screening, 2,963 studies were obtained. The number of publications on postpartum depression generally increased from 2000 to 2020, but increased more rapidly during the past 3 years (Figure 1).

### Analysis of Network Knowledge Map of National and Journals

**Figure 2:**
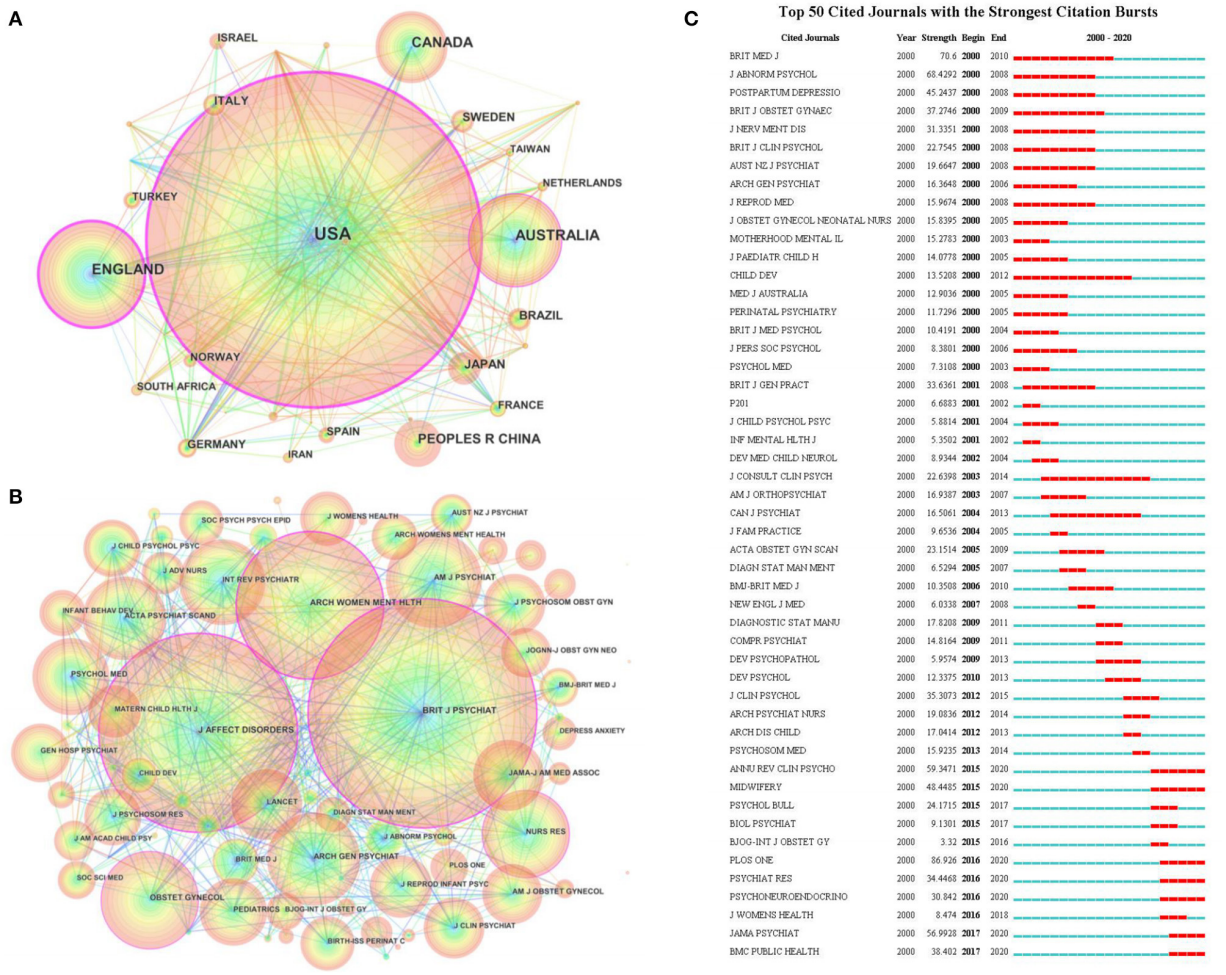
Co-occurrence analysis of countries and journals. **(A)** The countries co-occurrence network knowledge map of PPD from 2000 to 2020. **(B)** The journals co-citation network knowledge map of PPD from 2000 to 2020. **(C)** Top 50 cited journals with the strongest citation bursts.

From 2000 to 2020, 96 countries or regions published research associated with postpartum depression. Judging from the number of national publications (Figure 2A), the United States had the largest number of publications and ranked first with 1,047 articles and accounted for 35.33% (1,047/2,963) of the total—far exceeding the other countries. The countries ranked 2–10 were England, Australia, Canada, the People's Republic of China, Japan, Sweden, Brazil, Italy, Germany. The top 10 countries published 2,592 articles, which accounted for 87.47% (2,592/2,963) of the total. In terms of countries' betweenness centrality indicators, the United States, England, Australia, France, Norway, Austria, Germany, South Africa, Finland, Canada ranked among the top 10. The higher the betweenness centrality, the more important the country's research was recognized by other researchers. From the above results, the United States ranked first in terms of number and betweenness centrality, and was absolutely dominant in the field of postpartum depression.

A total of 717 journals published papers on postpartum depression between 2000 and 2020. After calculation, the Bradford dispersion coefficient was 7.328, the number of journals in the core area was 11.95, so our study showed that the top 12 journals published a total of 866 related papers, which accounted for 29.23% (866/2,963) of the total number of publications. Among them, the journal that published the most articles was the Archives of Womens Mental Health, which published 221 articles (Table 1) and accounted for 7.5% of the total (221/2,963). The journal was published by Springer Verlag. It was the most influential journal on postpartum depression (Reck et al., 2006). Its content involved psychodynamics social and biological aspects of all psychiatric and psychosomatic disorders in women. According to the betweenness centrality of the cited journals (Table 2 and Figure 2B), among the top 15 journals, eight journals were on psychiatry, three on obstetrics and gynecology, two on nursing, and two on pediatrics. From Figure 2C, seven journals —Annual Review of Clinical Psychology, Midwifery, Plos One, Psychiatry Research, Psychoneuroendocrinology, Jama Psychiatry, BMC Public Health paid more attention to the corresponding research on postpartum depression in recent years.

**Table 1:** Core journals of postpartum depression research published from 2000 to 2020.

**Rank**	**Journal**	**Publication n (%)**	**IF**
1	Archives of Womens Mental Health	221 (7.5)	2.5
2	Journal of Affective Disorders	201 (6.8)	3.892
3	Journal of Reproductive and Infant Psychology	74 (2.5)	1.188
4	Maternal and Child Health Journal	68 (2.3)	1.89
5	BMC Pregnancy and Childbirth	54 (1.8)	2.239
6	Jognn-Journal of Obstetric Gynecologic and Neonatal Nursing	44 (1.5)	1.25
7	Midwifery	39 (1.3)	1.778
8	Journal of Psychosomatic Obstetrics and Gynecology	36 (1.2)	2
9	Journal of Maternal-Fetal and Neonatal Medicine	34 (1.1)	1.737
10	Infant Behavior and Development	33 (1.1)	1.682
11	Journal Of Womens Health	32 (1.1)	2.104
12	BMC Psychiatry	30 (1.0)	2.704

**Table 2:** Ranking of the Top 15 cited-journals in the field of PPD by centrality from 2000 to 2020.

**Rank**	**Frequency**	**Betweenness Centrality**	**Journal**
1	2,272	0.37	British Journal of Psychiatry
2	2,011	0.33	Journal of Affective Disorders
3	1,476	0.21	Archives of Women Mental Health
4	1,022	0.11	Obstetrics and Gynecology
5	911	0.11	Nursing Research
6	910	0.08	Acta Psychiatrica Scandinavica
7	969	0.07	Archives of General Psychiatry
8	725	0.06	Pediatrics
9	660	0.06	Journal of Clinical Psychiatry
10	501	0.06	Journal of Advanced Nursing
11	243	0.06	Biological Psychiatry
12	1,031	0.05	American Journal of Psychiatry
13	674	0.05	Journal of Psychosomatic Obstetrics and Gynecology
14	479	0.05	Australian and New Zealand Journal of Psychiatry
15	397	0.05	Child Development

**Figure 3:**
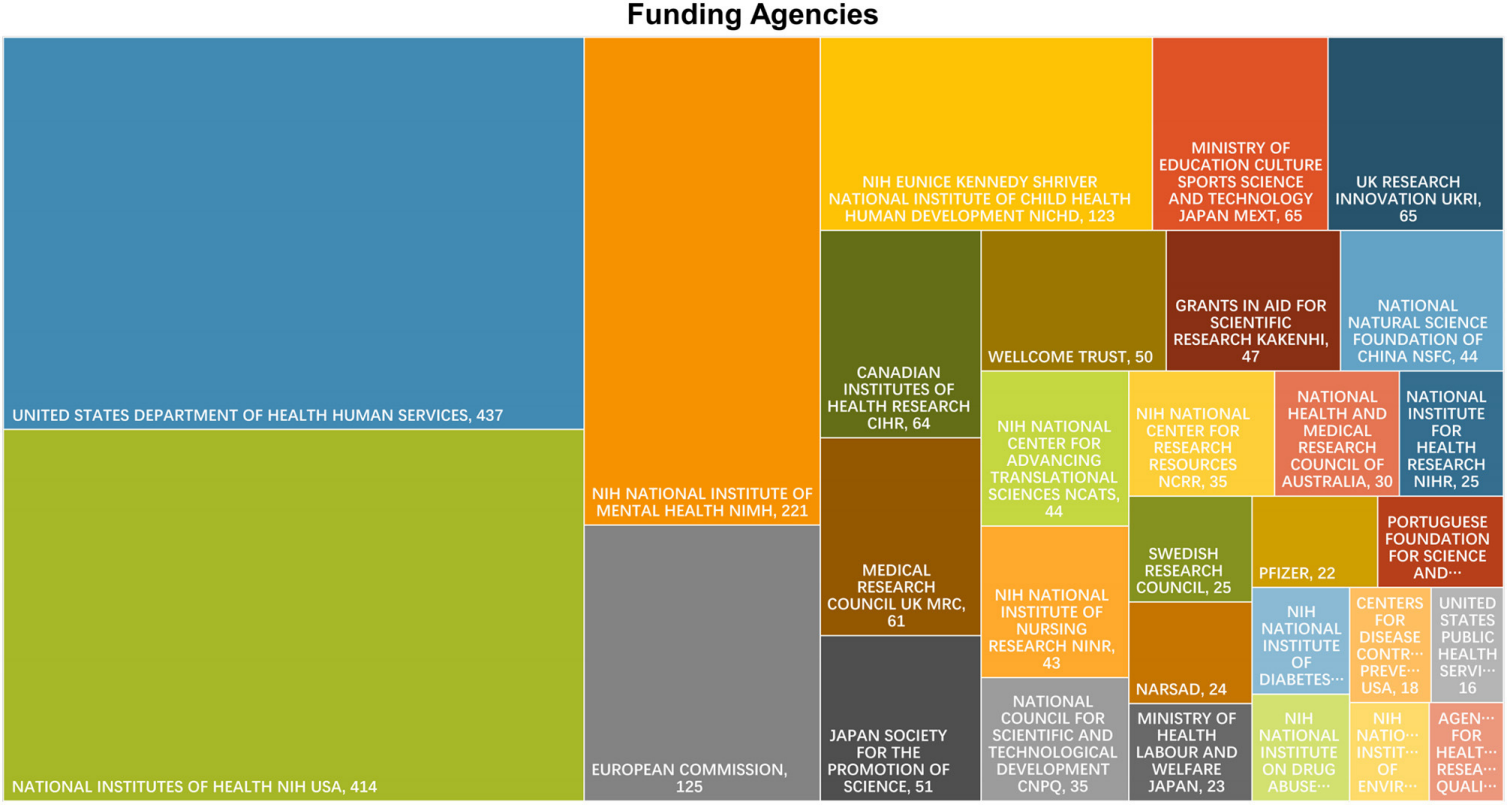
The Tree-map Visualization of funding agencies for literature on PPD.

In terms of funding institutions, U.S. funding agencies were dominated by United States Department of Health Human Services, National Institutes of Health NIH USA, NIH National Institute of Mental Health NIMH, NIH Eunice Kennedy Shriver National Institute of Child Health Human Development NICHD. The institute for UK Research Innovation UKRI and Medical Research Council UK were two of the largest funding agencies in the UK. Other national funding institutions were shown in Figure 3.

### Analysis of Research Hotspots Based on MeSH Terms Cluster

**Figure 4:**
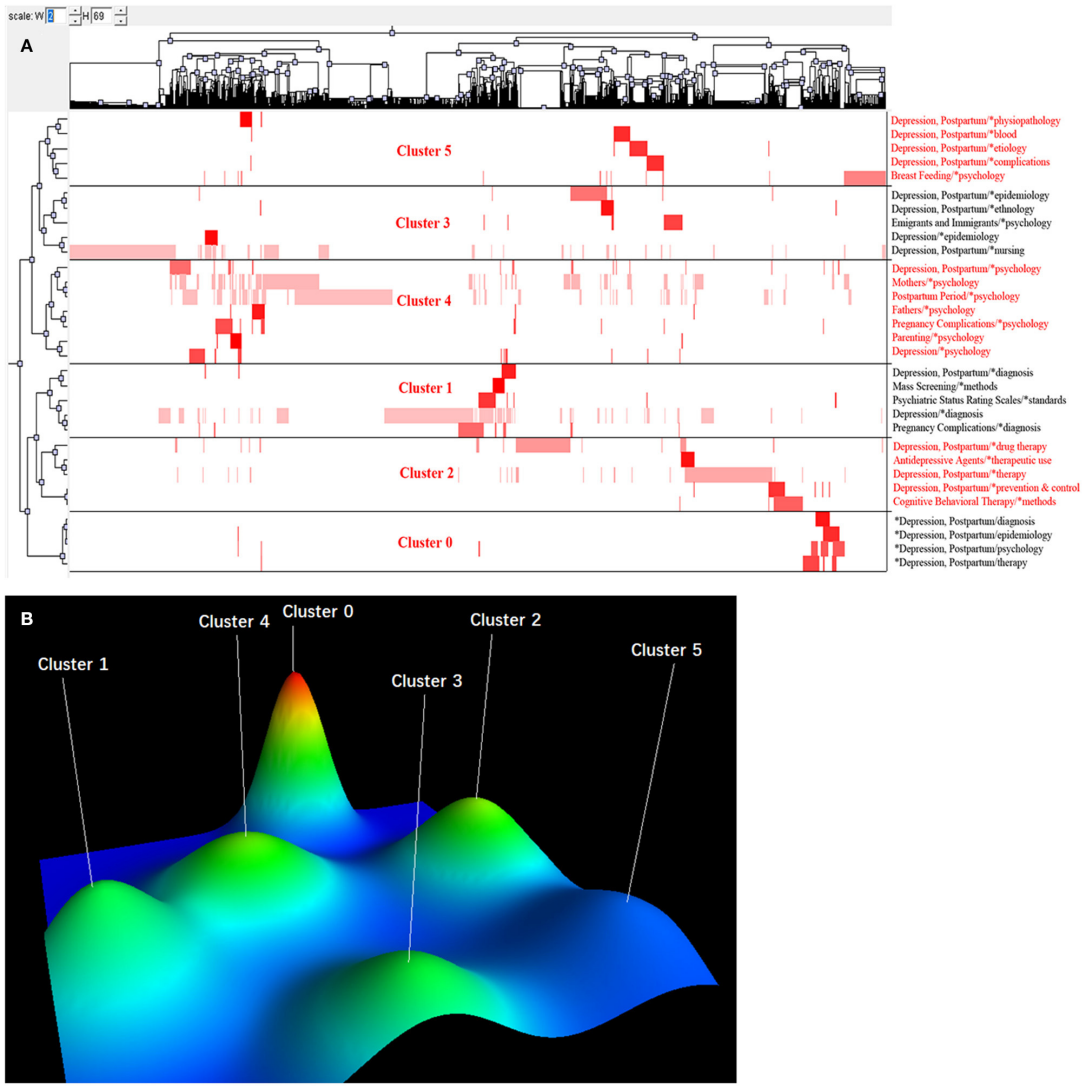
Biclustering analysis of 31 high-frequency main Medical Subject Heading (MeSH) terms/subheadings and articles on postpartum depression from 2000 to 2020. **(A)** Matrix visualization of biclustering of 31 high-frequency main MeSH terms/subheadings and PubMed Unique Identifiers of the articles. **(B)** Mountain visualization of biclustering of 31 high-frequency main MeSH terms/subheadings.

We used BICOMB to count 1,463 main MeSH terms/subheadings, and extracted 31 high-frequency main MeSH terms/subheadings (the cumulative percentage was 52.34%) (Table 3). Subsequently, we derived the high-frequency main MeSH terms/subheadings 31 rows × 2,811 columns of the co-word matrix. We imported the matrix into the gCluto software for a cluster analysis. According to the actual clustering effect, 31 high-frequency main MeSH terms/subheadings were clustered into six categories. The visualization matrix is shown in Figure 4A. The row of the visualization matrix was the clustering of 31 high-frequency main MeSH terms/subheadings. From the clustering results, there was little difference in the number of high-frequency main MeSH terms/subheadings in each cluster. The column was the clustering of the 2,963 articles that were displayed by the PMID number in the corresponding column at the top of the figure. The visual mountain map is presented in Figure 4B. From the overall layout, the clustering effect was better.

**Table 3:** High-frequency MeSH terms from the included papers on postpartum depression.

**Rank**	**Major MeSH terms/MeSH subheadings**	**Frequency**	**Proportion of frequency (%)**	**Cumulative percentage (%)**
1	Depression, postpartum/*diagnosis	466	6.7970	6.7970
2	Depression, postpartum/*psychology	456	6.6511	13.4481
3	Depression, postpartum/*epidemiology	449	6.5490	19.9971
4	Mothers/*psychology	373	5.4405	25.4376
5	Depression, postpartum/*therapy	257	3.7485	29.1861
6	Depression, postpartum/*prevention and control	186	2.7130	31.8991
7	Depression, postpartum/*etiology	131	1.9107	33.8098
8	Depression, Postpartum/*ethnology	102	1.4877	35.2975
9	Postpartum period/*psychology	93	1.3565	36.6540
10	Mass screening/*methods	88	1.2835	37.9376
11	Depression, postpartum/*drug therapy	79	1.1523	39.0898
12	*Depression, postpartum/diagnosis	72	1.0502	40.1400
13	Fathers/*psychology	65	0.9481	41.0881
14	Pregnancy complications/*psychology	62	0.9043	41.9924
15	*Depression, postpartum/epidemiology	58	0.8460	42.8384
16	Depression, postpartum/*nursing	56	0.8168	43.6552
17	Depression, postpartum/*physiopathology	49	0.7147	44.3699
18	Psychiatric status rating scales/*standards	49	0.7147	45.0846
19	Antidepressive agents/*therapeutic use	48	0.7001	45.7847
20	Depression, postpartum/*blood	47	0.6855	46.4702
21	*Depression, postpartum/psychology	45	0.6564	47.1266
22	Depression, postpartum/*complications	45	0.6564	47.7830
23	Depression/*diagnosis	40	0.5834	48.3664
24	Parenting/*psychology	38	0.5543	48.9207
25	*Depression, postpartum/therapy	37	0.5397	49.4603
26	Emigrants and immigrants/*psychology	36	0.5251	49.9854
27	Cognitive behavioral Therapy/*methods	35	0.5105	50.4959
28	Depression/*epidemiology	33	0.4813	50.9772
29	Breast feeding/*psychology	32	0.4667	51.4440
30	Pregnancy complications/*diagnosis	31	0.4522	51.8961
31	Depression/*psychology	31	0.4522	52.3483

Cluster 0 included four high-frequency main MeSH terms/subheadings. The height of the mountain was the highest and the color of the peak was red, indicating that the topic was similar and the distribution of articles was concentrated. The mountain was relatively independent and had poor relevance with the other five clusters. The cluster was an overview of studies related to postpartum depression.

Cluster 1 included five high-frequency main MeSH terms/subheadings. The height of the mountain was located in the middle, and the color of the peak was green, indicating that both the topic similarity and the distribution were in the middle. The cluster included studies related to the diagnosis of postpartum depression. The principal research revolved around postpartum depression screening, diagnostic methods, and postpartum depression assessment scale.

Cluster 2 included five high-frequency main MeSH terms/subheadings, and the color of the peak was green. The main focus was the treatment of postpartum depression, including treatment methods, cognitive behavioral therapy, treatment of postpartum depression complications.

Cluster 3 included five high-frequency main MeSH terms/subheadings, the color of the peak was light green, and the similarity within the cluster was in the middle. The cluster was linked to the epidemiology of postpartum depression. The core content was the risk factors and epidemiological characteristics of postpartum depression.

Cluster 4 included seven high-frequency main MeSH terms/subheadings, which were the largest in volume, was similar in height to Cluster 3, and the peak color was green. The cluster was related to the psychology of postpartum depression. The main focus was psychological changes in pregnancy complications or postpartum period, and effects of anxiety and stress on mothers.

Cluster 5 included five high-frequency main MeSH terms/subheadings, and the volume of the mountain were similar to Clusters 3. The peak color was blue indicating that the scope of research involving postpartum depression was loose and the literature was widely distributed. The cluster was related to etiology, physiopathology, complications, genetics of postpartum depression.

### Strategic Diagram Analysis

**Figure 5:**
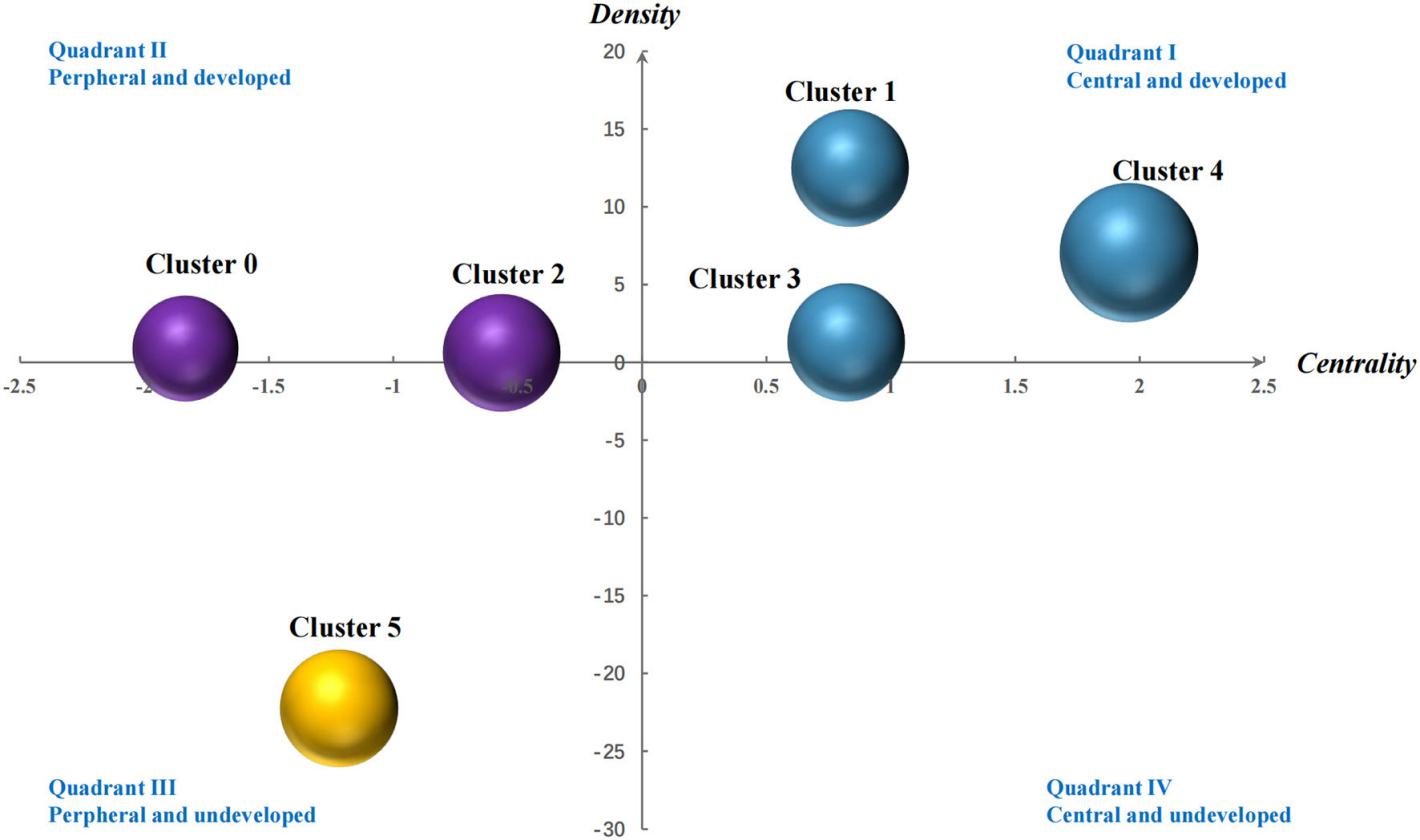
Strategic diagrams for postpartum depression–related research.

According to the results of the co-word analysis and the co-occurrence matrix of high-frequency main MeSH terms/subheadings, the centrality and density of each cluster were calculated. We used centrality and density to draw a two-dimensional strategic diagram (Figure 5). The research topics in Clusters 1, 3, and 4 had high centrality and density in the Quadrant I, indicating that the research on the diagnosis, epidemiology and psychology of postpartum depression was closely connected within the group, the research level was relatively mature, and the external connection had extensive connections with the other groups. They belonged to the core theme of postpartum depression. Cluster 0, 2 located in Quadrant II with high density and low centrality. It showed its research content was mature, but it had not been closely associated with other aspects of postpartum depression. Cluster 5, which was located in the Quadrant III, had the lowest centrality and density values, indicating that the research was broad and immature. Compared with other topics in the hot topics of postpartum depression research, they received relatively little attention and needed to be further developed.

## Discussion

With the development of modern social medicine, more attention has been paid to maternal mental health benefits. The modern medical model of “biology–psychology–society” requires attention not only to the physical health of puerpera, but also to the mental health level of puerpera. Known as the “hidden killer,” PPD is a depression disorder that occurs after childbirth and is the most common type of mental disorder in women. The pathogenesis of PPD is not completely clear, and may be related to genetic, neurobiochemical, and psychosocial factors. Due to the differences in social development level, cultural differences, and diagnostic criteria in different countries and regions, incidences of the disease vary greatly. At present, research on PPD mainly focuses on influencing factors (physiological, social, and delivery), diagnosis and treatment methods, and treatment measures (psychological intervention, drug therapy, and physical therapy) (Gao et al., 2007). With the increase in the global population and economic growth, PPD has attracted increasing attention. Thus, the number of published articles in major journals has increased gradually, especially in the last 3 years, with an exponential growth.

One of the research hotspots is the diagnostic and mental status rating scale of PPD, categorized as Cluster 1. The concept of PPD has expanded in the DSM-5, published by the American Psychiatric Association (APA) (Uher et al., 2014). The APA defines perinatal depression as depression occurring from the beginning of a pregnancy to the fourth week postpartum (Battle, 2013). Early screening is one of the keys to the prevention and treatment of PPD. Assessment scales are the main means of diagnosis, screening, and evaluation of PPD (ACOG, 2018); more than 24 types of depression screening scales are used during pregnancy (Bales et al., 2015). Among the many tools and strategies for identification, a rapid, inexpensive screening tool for perinatal women, the Edinburgh postnatal depression scale (EPDS), is the most widely applied (Cox et al., 1987). Its accuracy and psychometric properties make it one of the most established tools for depression screening in a range of perinatal populations. There are also the nine items of the patient health questionnaire (PHQ-9) and the Baker self-rating depression scale (BDI), which have good specificity and sensitivity, are concise and easy to operate, and are widely used across the world. The revised postpartum depression predictors inventory (PDPI-R), developed by Professor Beck in the United States, is used to screen psychosocial risk factors for postpartum depression (Beck, 2002; Beck et al., 2006) and is an assessment tool to predict the risk of postpartum depression. The predictive tools include 13 psychosocial risk factors for postpartum depression: marital status, socioeconomic status, self-esteem, prenatal depression, prenatal anxiety, unplanned pregnancy, depression history, social support, marital satisfaction, life stress, infant care stress, infant temperament, and maternal depression. The 39 items in the PDPI-R are divided into two parts, prenatal and postpartum, to predict the risk of postpartum depression during pregnancy and postpartum, respectively. The higher the score in the PDPI-R, the higher the risk of postpartum depression, and the Cronbach's α is 0.883. It has been promoted and applied in Italy (Grussu and Quatraro, 2009) and Japan (Ikeda and Kamibeppu, 2013). In addition, some studies have shown that one of the risk factors for PPD is genetic, but the genetic mode has not been determined. It was clear, however, that women with a family history of mental illness were significantly more likely to develop PPD (Tebeka et al., 2016; Bauer et al., 2018). Pregnant women who had previously been diagnosed with a clear mental disorder, especially depression, were at a higher risk of developing postpartum depression. People with a history of emotional disorder and premenstrual depression were also more susceptible to PPD (Yim et al., 2015).

In addition, the research center for PPD with mature research content is related to epidemiology and psychology, categorized as Cluster 3. The occurrence of PPD has psychosocial factors, such as negative life events, social support, psychological state during pregnancy, as well as age, ethnicity, educational level, and economic status. The relationship between age and PPD is controversial. Some studies believe that age has nothing to do with PPD, but some survey results show that the younger the maternal age, the higher the incidence of PPD, which may be related to the lower psychological maturity and weaker anti-stress ability of the younger maternal age. In addition, the relationship between literacy level and PPD is controversial. However, due to regional and cultural differences, economic and cultural levels, research methods, diagnostic criteria, and sample differences in different studies, the exact relationship between age and education level and PPD remains to be studied.

The mother–infant relationship in PPD, breastfeeding, and the psychological influence of parents, categorized as Cluster 4. Family relationships are one of the factors closely associated with PPD (Herzig et al., 2006). Domestic violence is associated with the severity of depression. High scores are positively correlated with domestic violence, as assessed by the EPDS (Bacchus et al., 2004). Studies have shown that pregnant women with an unstable personality are more prone to PPD, and according to the incidence of PPD, the personality of pregnant women can be sequentially ranked by type as: introverted–unstable, extroverted–unstable, extroverted–stable, and introverted–stable (López-Muñoz et al., 2019). Psychoanalysts believe that women become more primitive or childish in their behavior during pregnancy and after childbirth, and this change can cause psychological conflict. Due to the lack of understanding of the process of childbirth, excessive worry about the pain of childbirth, and the tension of childbirth, puerperium women are emotionally in a fragile state, and a week postpartum mood changes are more obvious. Excessive maternal anxiety and depression can lead to a series of physiological and pathological reactions and become a contributing factor to postpartum depression. In addition, the study found that there is an interaction between natural delivery, breastfeeding, early maternal and infant contact, and PPD.

There are various therapeutic for PPD, so the study of PPD intervention has become a new research hotspot (Cluster 2). Psychotherapy is the first-line therapy for postpartum depression and can make patients feel supported, respected, understood, and confident, strengthen self-control and the ability to establish good communication with others, stimulate the patient's intrinsic motivation to deal with their own problems, and has a significant effect on PPD. At the same time, it will not cause any danger to breastfed babies. Armstrong (Armstrong and Small, 2010) conducted a randomized, double-blind, controlled study of 181 women who were predisposed to PPD. Participants were assigned to either a nurse's home visit, a social worker's visit, a pediatrician's supportive intervention, or routine child-health services. After 6 weeks, women in the home-visit group had significantly lower PPD scores. In terms of the content of postpartum interventions, Chabrol's research shows that interventions consisting of four components, namely support, education, cognitive, behavioral, and psychological antagonism, can help to prevent, detect, and treat PPD (Chabrol et al., 2007). Stewart and Vigod (2019) reviewed the literature and selected 15 prospective controlled studies with a total sample size of 7,600, and concluded that intensive, professional postpartum support showed promising results. Current therapies include cognitive behavioral therapy (CBT), interpersonal psychotherapy (IPT), behavioral activation (BA), and mindfulness cognitive therapy (MBCT) (Dimidjian et al., 2016; Milgrom et al., 2016; Shulman et al., 2018; Loughnan et al., 2019; O'Hara et al., 2019). Second, there are also physical therapies, such as repetitive transcranial magnetic stimulation (rTMS), which causes local cortical function excitement or inhibition of the frontal cortex through rapid and repetitive TMS, so as to achieve the purpose of treatment. It is painless, non-invasive, and safe (Myczkowski et al., 2012). There are also antidepressants and traditional Chinese medicine for PPD, but there are also concerns about the side effects of drugs on offspring through breast milk (Li et al., 2016). In addition, drug therapy is recommended for patients who have no responded to psychotherapy or severe PPD, mainly including Selective Serotonin Reuptake Inhibitors (SSRIs) (preferred), and tricyclic antidepressants. But the medication can cause complications such as digestive disorders, sleep disturbances and irregular heart rhythms. Other drugs, such as estrogen, progesterone and melatonin, have therapeutic effects on PPD, but there are few clinical studies and no widespread application at present (López-Muñoz and Alamo, 2013; López-Muñoz et al., 2017; Lin et al., 2018). There is also VR virtual therapy, which is beneficial to help managing emotional problems of patients (Falconer et al., 2016; Yeung et al., 2021). For example, VR's distracting features can distract patients from the real-world situation (Eijlers et al., 2019), and the creation of presence function enables patients to “experiential” designed scenarios such as cognitive behavioral therapy (Riva et al., 2007; David et al., 2013).

At present, one of the areas that still needs to be studied further is the etiology and treatment of complications of PPD, especially the physiological pathology, which is Cluster 5. At present, the physiological causes of PPD are mainly neuroendocrine factors, which are considered to be abnormal central neurotransmitter metabolism and changes in related receptor functions, such as 5-hydroxytryptamine (5-HT), dopamine, and norepinephrine. In recent years, studies have focused on W-3 unsaturated fatty acids, such as eicosapentaenoic acid (EPA) and docosahexaenoic acid (DHA) (Markhus et al., 2015; Hsu et al., 2018; Mocking et al., 2020). Pawluski et al. (2017) also found through functional magnetic resonance imaging (fMRI) that the connections between the amygdala, anterior cingulate gyrus, dorsolateral prefrontal cortex, and hippocampus in PPD patients are significantly reduced, and the activity of the left amygdala is significantly reduced.

A review of bibliometrics articles related to depression reveals that researchers are paying more and more attention to psychological research as time goes by, such as the comorbidity of pain and depression (Wang et al., 2019), the links between the gut microbiota and depression (Zhu et al., 2020). However, there are few bibliometric studies on women's mental disorders (García-García et al., 2005; López-Muñoz et al., 2008), and almost no attention has been paid to the current research direction and hotspot of postpartum depression. Schanie et al. (2008) had analyzed the content of popular press magazine articles which focused on postpartum depression, published from 1998 to 2006, and 47 articles were identified in total. It proposed that health care providers should be proactive in directing childbearing women to factual sources of information on postpartum depression. Moreover, our research results show that the United States still accounts for the majority of the investment in PPD research, followed by the United Kingdom, which can be understood as developed countries pay more attention to the research on mental health and humanistic care. In addition, the number of Funding Agencies in Australia is lower than that in China, but the number of published papers is higher, which indicates that the effect of funding in Australia is significant.

In our study, the Web of Science core database was selected to improve the quality of literature inclusion, but the quality of the articles was mixed, and there might be some deviation in the research results. The current work searched WOS only which implying some papers could have been missed inevitably. But it would be impossible to merge multiple databases due to databases records with different reference counts. In this study, secondary information literature such as review and editorial were excluded to avoid repetition and interference of research hotspots. Because of the different selection of research methods and the lack of uniform standards for the limitation of the number of high-frequency terms, different scopes of inclusion of high-frequency terms may draw different conclusions, and at the same time, some emerging high-potential research directions may be filtered in recent years (Ding et al., 2001).

## Conclusion

In this study, the co-word clustering method was used for the first time to analyze related research on PPD, and comprehensively and objectively analyzed the research hotspots and research progress of PPD over the past 21 years. The number of researches is increasing scientifically. The etiology, physiological pathology, intervention, and treatment of complications on PPD are immature, which suggested that future studies should strengthen the study of PPD physiology, pathology, and genetics, and construct a comprehensive and dynamic patient management model and further explore the treatment of PPD.

## Data Availability Statement

The original contributions presented in the study are included in the article/supplementary materials, further inquiries can be directed to the corresponding author.

## Author Contributions

XB and DZ designed the study and drafted the manuscript. ZS and XW searched strategy. XB and YZ designed the statistical analysis plan, and DZ reviewed the manuscript. All authors take responsibility for the appropriateness of the content.

## Conflict of Interest

The authors declare that the research was conducted in the absence of any commercial or financial relationships that could be construed as a potential conflict of interest.
